# *In vitro* and *in vivo* Efficacy of a Synergistic Combination of Itraconazole and Verapamil Against *Aspergillus fumigatus*

**DOI:** 10.3389/fmicb.2019.01266

**Published:** 2019-06-07

**Authors:** Qiuqiong Zeng, Zheng Zhang, Peiying Chen, Nanbiao Long, Ling Lu, Hong Sang

**Affiliations:** ^1^Department of Dermatology, Jinling Hospital, School of Medicine, Nanjing University, Nanjing, China; ^2^Jiangsu Key Laboratory for Microbes and Functional Genomics, Jiangsu Engineering and Technology Research Center for Microbiology, College of Life Sciences, Nanjing Normal University, Nanjing, China

**Keywords:** *Aspergillus fumigatus*, verapamil, itraconazole, synergism, drug combination, bioluminescence imaging

## Abstract

The incidence of aspergillosis continues to rise sharply, while the progress made in expanding the antifungal drug arsenal remains extremely slow, indicating an urgent need for new strategies. Previous studies have shown that the calcium signaling pathway, which is evolutionarily conserved in mammals and fungi, is involved in regulating the tolerance of azoles in fungi. In this study, we performed a preliminary screening among various combinations of different clinical calcium channel blockers and different antifungal drugs. We found that the combination of itraconazole and verapamil showed the best synergistic effect against *Aspergillus fumigatus*. Thereafter, using the checkboard assays we observed synergistic effects of the combination treatment against most of the *A. fumigatus* strains tested, including itraconazole-sensitive and itraconazole-resistant strains, with a fractional inhibitory concentration index (FICI) < 0.5. Furthermore, we showed that verapamil strongly decreased the cytosolic calcium transients following itraconazole stimulation by an aequorin-mediated method. Moreover, verapamil influenced the efflux of rhodamine 6G, an azole mimic substance. An ergosterol assay revealed that verapamil alone had no effect on ergosterol biosynthesis, but the combination of itraconazole and verapamil treatment decreased the ergosterol level. Further murine assays were performed using a luciferase-probed bioluminescence imaging method. Drug combination therapy reduced lung burden and improved survival rate. In conclusion, verapamil is a promising candidate to enhance the antifungal activity of itraconazole against *A. fumigatus*. In addition, our study suggests the effectiveness of an emerging approach based on bioluminescence imaging in monitoring the efficacy of drug combination therapy for invasive aspergillosis.

## Introduction

*Aspergillus fumigatus* is a ubiquitous and opportunistic filamentous fungal pathogen that can cause invasive, chronic, and allergic aspergillosis. Invasive aspergillosis is one of the most important life-threatening fungal infections and mainly affects immunocompromised hospitalized patients, such as patients with hematological malignancies or AIDS and solid organ or hematopoietic stem-cell transplant (HSCT) recipients (Morgan et al., 2005; Rubio et al., 2009; Taccone et al., 2015; Koehler et al., 2017; Zilberberg et al., 2018). This condition has a very high mortality rate ranging from 40 to 90% (Montagna et al., 2013). Currently, there are only three categories of antifungals used in aspergillosis treatment: polyenes, azoles, and echinocandins. Azoles such as itraconazole (ITC) and voriconazole (VRC), which target 14-alpha-lanosterol demethylase, a crucial enzyme in the ergosterol biosynthesis pathway, are the first-line recommended options for prevention and treatment of aspergillosis due to their fewer side effects and broader antimicrobial spectrum compared to polyenes and echinocandins (Patterson et al., 2016). However, antifungal therapy remains a major challenge because of the insufficient therapeutic options, drug toxicity, inter-individual variation, and most importantly, the appearance and widespread prevalence of azole-resistant isolates (Liu et al., 2015b; Hagiwara et al., 2016; Chowdhary et al., 2017; Fisher et al., 2018). Long-term administration of azoles to susceptible populations for prevention or treatment results in natural selection of resistant isolates since the azoles are fungistatic rather than fungicidal (Campoy and Adrio, 2017). The strategies of azole tolerance adopted by fungi (Sanglard, 2016; Rybak et al., 2018) include (i) drug target alterations, (ii) reduction of effective cellular drug concentration, and (iii) stress adaptation by modifying the cellular metabolic pathway. Many researchers have searched for alternative drug targets or tried to develop safe broad-spectrum antifungals (Wiederhold et al., 2015; Arendrup et al., 2016; Colley et al., 2018). However, eukaryotic cells, including fungal cells and their eukaryotic host cells, share evolutionarily conserved molecular signaling pathways, which limit the fungal-specific candidates that can be targeted by new drugs. Furthermore, development of novel antifungal agents is very costly and time-consuming (Campoy and Adrio, 2017). Thus, mining existing agents that can enhance the efficacy of antifungal drugs is a promising approach to improve the drug susceptibility of *A. fumigatus*.

The calcium signaling pathway plays an important, wide ranging physiological role in all organisms. In this pathway, the putative plasma membrane Ca^2+^ influx system of *A. fumigatus* is the CchA-MidA complex protein, which is homologous to human L-type voltage-gated calcium channels. The CchA-MidA complex mediates a rapid influx of calcium ions and leads to transient increases in intracellular calcium concentrations, which affect a wide range of essential cellular processes. Azoles were shown to upregulate the mRNA levels of the *cch* and *mid* genes in *Candida albicans* and *A. fumigatus* (Gamarra et al., 2010; Liu et al., 2015a). Disruption in ergosterol synthesis by azole treatment resulted in calcium dependence in *Saccharomyces cerevisiae* (Crowley et al., 1998). Thus, agents that interfere with calcium balance are involved in the regulatory mechanism for fungal stress adaption under azole environments in fungi (Edlind et al., 2002; Liu et al., 2015a,c). Calcium channel blockers (CCBs) are clinical commonly used class IV antiarrhythmia agents, which target the L-type voltage-gated calcium channels in mammalian cells, are possible candidates to interfere with Ca^2+^ entry in fungi and attenuate the metabolic modification of fungal stress adaption and potentiate the antifungal effect of azoles. However, little is known about whether clinical CCBs could enhance the *in vitro* and *in vivo* antifungal activity of azoles against *Aspergillus*.

In this study, after a preliminary screening of different CCBs and antifungals, we showed that verapamil (VER) displayed a synergistic effect with ITC against an *A. fumigatus* strain *in vitro* and *in vivo*. To monitor the disease progression during drug therapy, a luciferase-proved *A. fumigatus* strain was constructed as a reporter strain to realize real-time tracking.

## Materials and Methods

### Strains, Culture Conditions, and Compounds

The strains used in this study mainly included the *A. fumigatus* strains A1160 (Δ*ku80 pyrG1*, from Fungal Genetics Stock Center), Af1161 (Δ*ku80* A1160::*pyrG1*) (Song et al., 2016), Af1160-AEQ (Δ*ku80 pyrG1;pAEQcyt*) (Song et al., 2016) and Af1161-Luc2 (Δ*ku80* A1160::*pyrG1;pLUC*) (constructed in this study). The molds were refreshed from the frozen storage at -80°C and cultured on YAG solid medium (containing 0.5% yeast extract, 2% glucose, 2% agar, and 0.1% trace element solution) or YUU solid medium (containing YAG plus 10 mM uracil and 5 mM uridine) at least twice at 37°C for 2–3 days before each experiment to ensure viability and purity. Spores were harvested and adjusted to the concentration needed. Drugs used *in vitro* were obtained as powders. ITC was provided by Xi’an Janssen-Cilag Company, and a stock solution of 10 mg/ml was prepared in dimethyl sulfoxide (DMSO). Pure VER, nifedipine, diltiazem, voriconazole, and amphotericin B were purchased from the Chinese Medicine Institute. VER, nifedipine and diltiazem were prepared in DDW, and a stock solution of 10 mg/ml was prepared. VRC and amphotericin B were diluted in DMSO. All stock solutions were stored at -20°C.

### Susceptibility Testing and Checkboard Assay for Interaction Evaluation

Determination of the drug susceptibility was performed using checkboard assay based on the CLSI M38-A2 protocol (Wayne, 2008). Briefly, serial ITC (fourfold) dilutions (50 μl per well) were prepared in multiwell plates with the presence or absence of a fourfold concentration of VER (50 μl per well). The final concentration of ITC ranged from 0.0313 to 16 μg/ml, and VER ranged from 10 to 9600 μg/ml. Drug-free wells were used as controls. Freshly isolated spores suspended in 100 μl RPMI 1640 medium were added to each well. After incubation for 48 h at 35°C, the MIC was determined as the lowest concentration showing no discernible growth. The interactions were determined by fractional inhibitory concentration index (FICI) values calculated by the following equation:

$$\text{FICI}\;=\;\frac{\text{MIC}\;\left(\text{Acomb}\right)}{\text{MIC}\;\left(\text{Aalone}\right)}\;+\;\frac{\text{MIC}\;\left(\text{Bcomb}\right)}{\text{MIC}\;\left(\text{Balone}\right)},$$

where MIC (Aalone) and MIC (Balone) represent the MIC value of drug A and drug B used alone, respectively, and MIC (Acomb) and MIC (Bcomb) represent the MIC value of drug A and drug B with combined use, respectively. FICI was interpreted as follows: FICI ≤ 0.5 indicates a synergistic effect; 0.5 < FICI ≤ 4 indicates no interaction; and FICI > 4 indicates an antagonistic effect. Each experiment was performed in triplicate on three different days.

### Cytoplasmic Ca^2+^ Measurement

The real-time monitoring of cytosolic free calcium concentrations ([Ca^2+^]_c_) was performed by the method described previously with minor modifications (Zhang et al., 2016). Af1160-AEQ, which carried the codon-optimized aequorin gene, was cultured in liquid MM in white 96-well plates at 37°C for 18 h away from light. The mycelia were washed with 200 μl PGM (20 mM PIPES, 50 mM glucose, 1 mM MgCl_2_, pH 6.7) twice and then incubated in 100 μl PGM containing 2.5 μM aequorin cofactor coelenterazine (Sigma-Aldrich) at 4°C for 4 h in the dark. After the samples were washed with PGM twice, the mycelia were incubated at room temperature for 1 h to recover their activity. Before stimulus injection, 1 mM EGTA or 1 mM VER was added into each well. The luminescence was recorded for a total period of 180 s, while 1 μg/ml ITC or 1 mM VER was added at 20 s, and after that, the active aequorin was discharged completely by 20% ethanol containing 3 M CaCl_2_ to calculate the total aequorin light emission of each well. The machine used in this study was an LB 96 Microlumat luminometer (Berthold Technologies, Germany). Finally, the relative luminescence units (RLUs) were recorded and converted into [Ca^2+^]_c_ as described previously. For each treatment, six wells were conducted in parallel. Each experiment was repeated at least three times.

### Measurement of Rh 6G Uptake and Efflux Abilities

The measurement of the azole-mimicked substrate rhodamine 6G (Rh 6G) was performed as described by Wei et al. (2017) with slight modifications. Briefly, approximately 5 × 10^6^ fresh spores were suspended in 1 ml YAG with or without VER and vortexed for 5 min. The suspension was transferred to 35 mm petri dishes and incubated at 37°C for 4 h, when the conidia began to germinate. Thereafter, the mixture was gently washed three times with phosphate-buffered saline (PBS). Then, the samples were suspended in PBS with Rh 6G at a final concentration of 10 μM and incubated for 1 h at 37°C. The samples were washed and suspended in PBS, and fluorescence was measured for 10,000 conidia using a fluorescence-activated cell sorter (FACS) analysis system (Accuri C6; BD). For determination of ATP-dependent R6G efflux, 10% glucose was added to the sample and incubated for 30 min.

### Ergosterol Extraction and Analysis

The extraction of total ergosterol from *A. fumigatus* mycelia was performed with minor modifications as described previously (Long et al., 2017). Briefly, approximately 1 × 10^8^ spores were incubated in 100 ml liquid media with relevant concentrations or drugs for 24 h at 37°C at a speed of 220 rpm. Mycelia were harvested via filtration and washed with distilled water. Then, 200 mg lyophilized mycelia were treated with 3 ml of 25% alcoholic potassium hydroxide (methanol to ethanol, 3:2), vortexed for 1 min, and incubated for 1 h at 85°C. After the samples cooled to room temperature, they were combined with 3 ml of pentane and 1 ml of distilled water and then vortexed for 3 min. After the samples stood still for 10 min, the upper hexane layer was transferred to a new tube. Then, the samples were evaporated in a fume hood and dissolved in 1 ml of methanol. After filtration through a pore-size 0.2 μm filter, the samples were analyzed by high-performance liquid chromatography (HPLC) (Agilent Technologies) with an AQ-C_18_ column (250 mm by 4.6 mm, 5 μm) for 15 min at 282 nm with a flow rate of 1 ml/min.

### Live Animal Bioluminescence Imaging

The *A. fumigatus* isolate Af1161 was transformed with luciferase reporter genes with the *gpdA* promoter as previously described with minor modifications (Brock et al., 2008; Ding et al., 2013). Spores were obtained and suspended in PBS. The activity was confirmed using microplate reader (Spectra Max M2) and IVIS Lumina XR system (PerkinElmer) with a spore suspension containing 1% luciferase reporter reagent (Promega). Infected mice were introduced intraperitoneally with 100 μl D-luciferin in PBS (33.3 mg/ml) 10 min before detection. Mice were anesthetized using 2.5% isoflurane with a constant flow and then placed in an IVIS Lumina XR system chamber. Scanning was performed on days 2, 4, and 6. The region was cropped in the chest area from each animal, and the total bioluminescence signal intensity of the lung region was extracted and analyzed using Living Image 4.2 (Caliper Life Sciences).

### Animal Infection and Histopathology

Animal infection assays were performed in accordance with the method described previously, with slight modifications (Galiger et al., 2013; Zhang et al., 2015). Briefly, BALB/c male mice (8 weeks old, 23–28 g) were immunosuppressed on days -4 and -1 with 150 mg/kg cyclophosphamide. On day 0, mice were infected by tracheal cannula with a suspension containing 5 × 10^5^ conidia or PBS as the control in a total volume of 50 μl after anesthetization with 1% pentobarbital sodium (10 ml/kg weight). After they were completely awake, the mice were administered the relevant drugs via gavage for 6 days. In order to observe potential synergy, we used a suboptimal dose of ITC (20 mg/kg), lower than the human bioequivalent dose in mice of ITC (30 mg/kg). For VER, the human bioequivalent dose in mice (5 mg/kg) was selected. For maintenance of immunosuppression, 75 mg/kg weight cyclophosphamide was injected every 3 days after infection. For reduction of animal distress, the body weights of the mice were recorded daily, and when they lost 20% of their initial weight, mice were euthanized. Mice were examined daily for 14 days. Histology of lung tissue was processed, and Grocott’s methenamine silver stain and periodic acid-Schiff (PAS) staining were performed.

This study was carried out in accordance with the recommendations of the Guide for the Care and Use of Laboratory Animals of the National Institutes of Health, United States. The protocol was approved by the Animal Care and Use Program at Nanjing Normal University, China (protocol number 2090658).

### Statistical Analysis

The results were statistically analyzed using the software IBM SPSS Statistics 23. Student’s *t* test was used for statistical analysis. The log-rank (Mantel-Cox) test was performed for survival analysis. A *p*-value < 0.05 was considered significant.

## Results

### Preliminary Screening of Clinical CCBs Enhancing the Antifungal Effect of Antifungal Drugs

**Figure 1:**
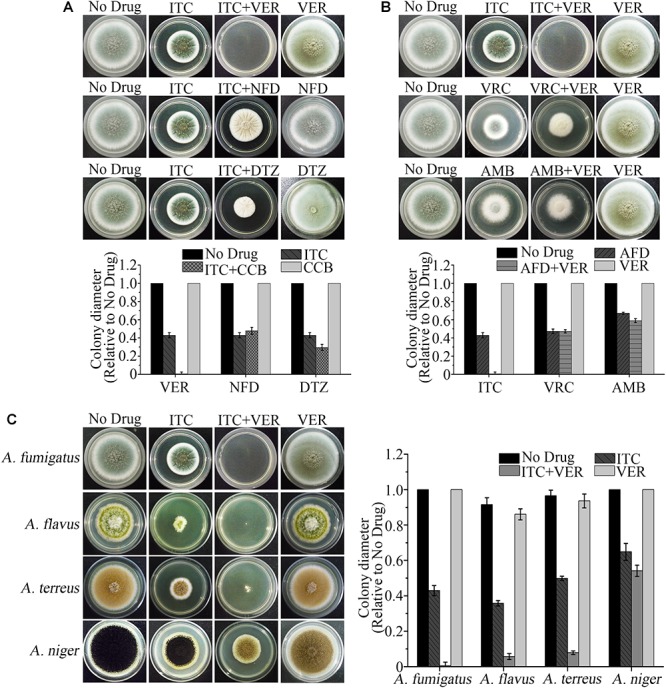
The ITC-VER combination is the most promising candidate among antifungal drugs and calcium channel blockers (CCBs) against *Aspergillus* spp. **(A)** Testing the growth inhibition on agar plates by itraconazole (ITC) (0.1 μg/ml) with different CCBs, including verapamil (VER), nifedipine (NFD) and diltiazem (DTZ), at 200 μg/ml against an *A. fumigatus* stain. **(B)** Testing the growth inhibition on agar plates by VER (600 μg/ml) with different antifungal drugs (AFD), including ITC (0.1 μg/ml), voriconazole (VRC) (0.05 μg/ml) and amphotericin B (AMB) (1.5 μg/ml), against an *A. fumigatus* stain. **(C)** Testing the growth inhibition on agar plates by the ITC-VER combination against *A. fumigatus* (0.1 and 200 μg/ml, respectively), *A. flavus* (0.04 and 50 μg/ml, respectively), *A. terreus* (0.1 and 50 μg/ml, respectively) and *A. niger* (0.1 and 600 μg/ml, respectively).

To find the drug combination that displays the best synergism, we initially performed a preliminary screening by comparing the antifungal effects among three representative CCBs and three commonly used antifungal drugs against the clinical *A. fumigatus* isolate Afc03. As shown in Figure 1A, with the same concentration of ITC (0.1 μg/ml), the addition of VER, the representative phenylalkylamine among the clinical antiarrhythmia agents, significantly enhanced the antifungal activity of ITC, resulting in an obvious reduction in colony diameter on agar plates compared to that of nifedipine and diltiazem, the representative dihydropyridine and benzodiazepine CCBs, respectively, at the same concentration as VER. In contrast, no detectable antifungal effects were found in the presence of the same concentrations of VER, nifedipine, and diltiazem alone. No differences in colony diameter were detected with VRC or amphotericin B at a subinhibitory concentration with the addition of the same concentration of VER compared to VRC or amphotericin B alone (Figure 1B). To test whether the synergistic effect of the combination of ITC and VER can be applied to other important *Aspergilli*, including *A. flavus*, *A. terreus*, and *A. niger*, which caused aspergillosis, we further tested the effects of this treatment on various clinical strains. As shown in Figure 1C, the combination of VER and ITC also reduced the diameters of the colonies of *A. flavus* and *A. terreus*, but no differences were observed in *A. niger* with the combination compared to ITC alone. These preliminary screening results suggested that the combination of VER and ITC was the best candidate to show a synergistic effect against *A. fumigatus* strains among the representative CCBs and antifungal drugs tested.

### Selective Synergistic Inhibitory Effect of VER and ITC Against Susceptible and Resistant *A. fumigatus*

To further determine the antifungal activities of ITC and VER, either alone or combined, against *A. fumigatus*, we tested 17 *A. fumigatus* isolates, including 6 ITC-susceptible (ITC-S) and 11 ITC-resistant (ITC-R) strains, using the checkboard technique, a two-dimensional microdilution method based on CLSI M38-A2, the document about antifungal susceptibility testing for filamentous fungi. As shown in Table 1, for the quality control strain ATCC MYA-3626, the MIC value of ITC, based on no growth, was 0.25 μg/ml, which is within the reference range. For VER, no reference range has been established. Nevertheless, VER within the drug range tested appeared to exhibit no antifungal effect against all *A. fumigatus* isolates tested. The MIC values for ITC-S isolates were 0.25–0.5 μg/ml, and those for ITC-R isolates were 2–16 μg/ml.

**Table 1:** Antifungal susceptibility testing and drug interaction evaluation between ITC and VER against *A. fumigatus*.

Strain	MIC (μg/ml)				
	Alone		Combined		ITC-VER interaction	
	ITC	VER	ITC	VER	FICI	Interpretation^a^
Afc03^‡^	0.25	> 9600	0.0625	40	< 0.254	SYN
Afc06^‡^	0.25	> 9600	0.0625	40	< 0.254	SYN
Afc07^‡^	0.25	> 9600	0.0625	80	< 0.258	SYN
Afc18^‡^	0.5	> 9600	0.125	80	< 0.258	SYN
Af1161	0.25	> 9600	0.0625	80	< 0.258	SYN
Af293	0.25	> 9600	0.25	80	< 1.008	NI
Shjt40^†^	8	> 9600	1	960	< 0.225	SYN
Shjt42b^†^	16	> 9600	16	960	< 1.1	NI
Afc3-339	16	> 9600	4	480	< 0.3	SYN
Afc6-75	2	> 9600	0.25	480	< 0.175	SYN
Afc7-233	16	> 9600	16	960	< 1.1	NI
Af1161-73	16	> 9600	4	480	< 0.3	SYN
Af1161-132	8	> 9600	2	960	< 0.35	SYN
Af1161-78	16	> 9600	2	960	< 0.225	SYN
Af1161-185	16	> 9600	4	480	< 0.3	SYN
Af1161-331	16	> 9600	2	960	< 0.225	SYN
Af1161-353	16	> 9600	8	960	< 0.6	NI

^a^*Abbreviations: FICI, fractional inhibitory concentration index; ITC, itraconazole; NI, no interaction; SYN, synergism; VER, verapamil. ^‡^Clinical-derived isolates susceptible to itraconazole. ^†^Clinical-derived isolates resistant to itraconazole (8)*.

For the ITC-S strains with combination treatment, the FICI values were 0.254–0.258, which indicated significant synergism, in all ITC-S isolates from clinical sources (including the Afc03, Afc06, Afc07, and Afc18 strains) and the ITC-S isolate Af1161, a laboratory wild-type strain derived from the clinic. In contrast, the combination displayed no interaction against another laboratory-derived ITC-S isolate, Af293 (FICI = 1.008). These data showed that cotreatment with VER improved ITC susceptibility in most ITC-susceptible strains. Meanwhile, for ITC-R isolates, a synergistic effect was observed in 8 of 11 ITC-R isolates, with FICI values of 0.175–0.35. In comparison, no interaction was observed in three laboratory-induced ITC-R isolates (FICI range of 0.6–1.1). These observations indicated that tolerance to ITC of resistant *A. fumigatus* may be lost in the presence of VER in some strains. The results of growth inhibition on agar plates were consistent to the checkboard results (see Supplementary Figure S1).

We concluded that VER had poor antifungal activity against *A. fumigatus* but acted as a potential sensitizer of ITC in all clinically derived ITC-S *A. fumigatus* strains tested in this study, but no specific rule was found for ITC-R strains from clinical and laboratory-derived sources.

### The Cytosolic Ca^2+^ Signature Induced by ITC Is Remarkably Reduced by VER in *A. fumigatus*

VER is a known L-type voltage-gated CCB in mammals. To determine whether the effect of blocking the calcium signaling pathway by VER was also present in fungi, we performed routine measurements of cytosolic free Ca^2+^ ([Ca^2+^]_c_) dynamics in living spores using the Af1160-AEQ strain, which carried the aequorin reporter gene. The results of fungal growth in solid culture or FICI measured by the checkboard method were identical between the Af1160-AEQ strain and the parental wild-type strain Af1160 (see Supplementary Table S1).

**Figure 2:**
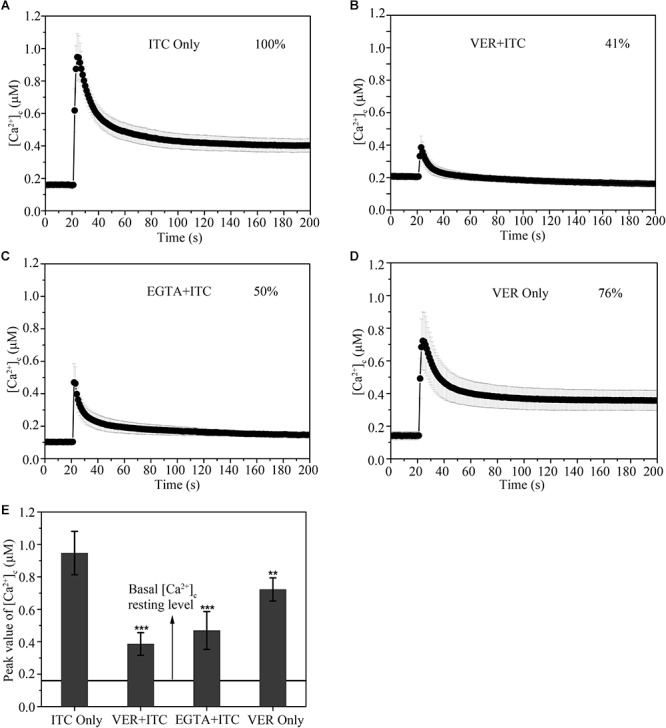
Verapamil blocks the [Ca^2+^]_c_ transient induced by cellular membrane pressure following itraconazole. **(A–D)** [Ca^2+^]_c_ responses to ITC (1 μg/ml) stimulation **(A)** pretreated with 1 mM VER **(B)** or 1 mM Ca^2+^-chelator EGTA (C) for 10 min. [Ca^2+^]_c_ responses to VER (1 mM) stimulation **(D)**. **(E)** The peak concentrations of [Ca^2+^]_c_ are shown in the bar graph, relative to the group with ITC stimulation, ^∗∗^*p* < 0.01, ^∗∗∗^*p* < 0.001. The prestimulatory basal [Ca^2+^]_c_ resting level, indicated by the line, is approximately 0.16 μM. In each experiment, average values of six independent replicates are shown with SD error bars.

The resting level of [Ca^2+^]_c_ was approximately 0.18 μM in the untreated sample (Figure 2D). After stimulation with 1 μg/ml ITC, there was a significant increase in [Ca^2+^]_c_ up to ∼0.95 μM, followed by a pronounced decrease in [Ca^2+^]_c_ to approximately 0.6 μM, and the level steadily decreased to 0.4 μM until the end of the experiment (Figure 2A). The resting level of poststimulatory [Ca^2+^]_c_ showed a > onefold increase relative to the prestimulatory resting level in the untreated sample following ITC exposure, suggesting a disturbance in the return of [Ca^2+^]_c_ to its normal resting level. The initial large transient [Ca^2+^]_c_ increases in response to ITC were substantially inhibited after pretreatment with 1 μM of the Ca^2+^ channel blocker VER, although the [Ca^2+^]_c_ resting level was generally increased (approximately 0.2 μM) (Figure 2B). The maximum [Ca^2+^]_c_ amplitude following VER pretreatment was 41% of that of the untreated samples. In contrast, pretreatment with EGTA, a Ca^2+^-chelator, also significantly reduced the amplitude of the [Ca^2+^]_c_ (50% relative to untreated samples) in response to 1 μg/ml ITC (Figure 2C). The amplitude of the [Ca^2+^]_c_ was decreased by 76% after VER stimulation compared to ITC stimulation (Figure 2D). These results indicated that VER, as a CCB in mammalian cells, can decrease the Ca^2+^ signal induced by ITC in *A. fumigatus*, which influences the subsequent response of the calcium channel pathway for fungal survival.

### Pretreatment With VER Decreases the Efflux of Rh 6G in *A. fumigatus*

**Figure 3:**
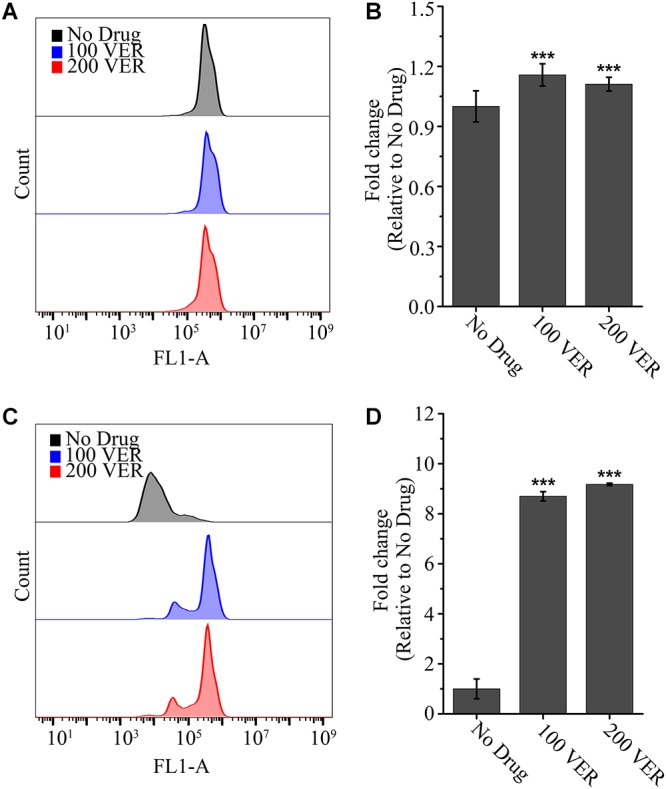
Pretreatment with verapamil increases the efflux of Rh 6G. The uptake **(A)** and efflux **(C)** of Rh 6G were assayed with or without pretreatment of 100 or 200 μg/ml VER in an *A. fumigatus* strain by flow cytometry analysis. FL1-A represents the relative fluorescence intensity value. Normalized quantification of the fluorescence intensity is shown in bar graph **(B,D)** relative to that of the No Drug group. Values represent the mean ± SD of three replicates. ^∗∗∗^*p* < 0.001.

To further assess whether the drug efflux pump function is influenced by VER, we examined the activity of the drug efflux pump with or without VER preincubation using the azole-mimicking substrate Rh 6G, a tracer dye. As shown in Figure 3, no detectable difference was found in the uptake of Rh 6G between the untreated and VER-pretreated groups (Figure 3A,B). However, in the glucose-induced efflux assay, we observed more retention of Rh 6G with VER pretreatment at the same concentration than that of the untreated group. Approximately 8.7-fold and 9.2-fold increases in the mean fluorescence intensity (FLA-1) compared to that of the untreated group were observed in the 100 μg/ml VER and 200 μg/ml VER groups, respectively (Figure 3C,D). We concluded that VER attenuated the ability to pump out the azole-mimic substrate Rh 6G with ATP-driven drug efflux, resulting in an increase in Rh 6G retention. These data demonstrated that the ability to block the drug pump function of VER plays a direct role in the synergistic antifungal activity of the ITC-VER combination.

### VER Decreases the Ergosterol Content Combined With ITC, but VER Alone Displays No Effect on Ergosterol Biosynthesis

**Figure 4:**
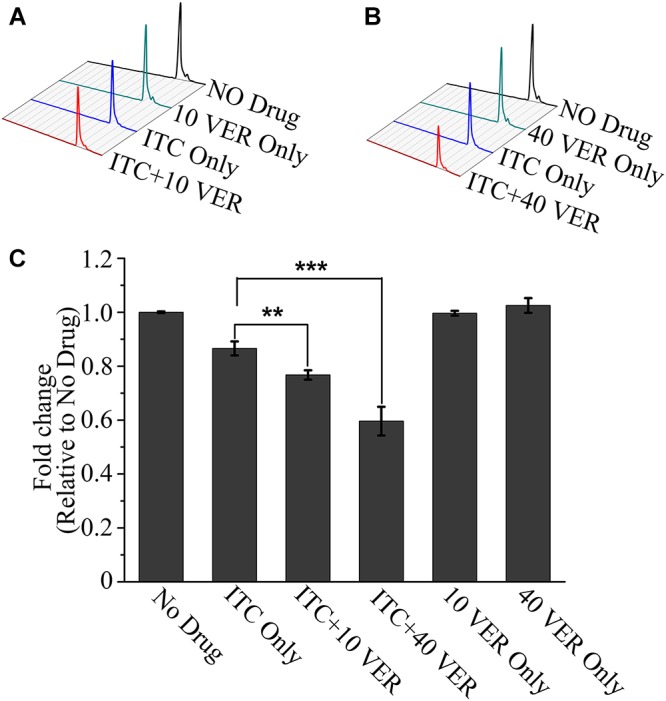
HPLC assay showing a decrease in ergosterol in the ITC-VER combination. **(A,B)** Ergosterol was extracted from the *A. fumigatus* strain treated with 0.02 μg/ml ITC and 10 μg/ml **(A)** or 40 μg/ml **(B)** VER for 24 h and then measured by HPLC. **(C)** Normalized quantification and comparison of ergosterol contents. The bar graph is shown as a percentage relative to the No Drug group. Error bars in the graph represent standard deviations from triplicate experiments. ^∗∗^*p* < 0.0l; ^∗∗∗^*p* < 0.001.

Previous studies have demonstrated that azoles can disturb the synthesis of 14-α-lanosterol demethylase, which is crucial for the biosynthesis of ergosterol (Chowdhary et al., 2017). To further investigate the synergistic mechanism of the ITC-VER combination, we compared the ergosterol contents following different drug treatments. As shown in Figure 4, the ergosterol level determined after VER alone showed no difference from that of the untreated group. In contrast, after treatment with ITC alone at a low concentration, 13% of the ergosterol content was lost compared to that of the untreated group. Nevertheless, in the presence of the same concentration of ITC, the addition of 10 μg/ml VER or 40 μg/ml VER resulted in a significant decrease in the ergosterol content, with ratios of 0.78 and 0.57, respectively, compared to that of the No Drug group (Figure 4C). These observations demonstrated that VER does not affect ergosterol biosynthesis on its own, but the ergosterol inhibition of ITC was improved by adding VER in *A. fumigatus*.

### An *in vivo* Mouse Model Demonstrates the Synergistic Effect of VER and ITC

As mentioned in the *in vitro* study, VER displayed a synergistic effect with ITC against *A. fumigatus* in growth inhibition on agar plates and checkboard assays. We wondered whether a synergistic effect existed under *in vivo* conditions. To test this hypothesis, we constructed a bioluminescent reporter strain, Af1161-Luc2, with an introduced codon-optimized luciferase-probed plasmid in which luciferase expression is driven by the *A. fumigatus gpd* promoter (*gpd*-luciferase) to follow the disease progression and quantify the fungal burden in the lung. The reporter activity was verified by luciferase enzyme using a microplate reader and living imaging (see Supplementary Figure S2). To evaluate the drug susceptibility of Af1161-Luc2 compared to its parental wild-type strain, we further detected the *in vitro* activity of this strain. No significant differences were observed between the parental strain Af1161 and the reporter strain Af1161-Luc2 in MIC or FICI values (see Supplementary Table S1). Subsequently, immunocompetent mice were challenged with the Af1161-Luc2 strain or the Af1161 strain as a control, directly by the trachea, to resemble the natural respiratory route of invasive pulmonary aspergillosis most closely, followed by administration of different drugs alone or combined. The progression of infection or recovery of mice experiencing pulmonary disease was determined by body weight measurement and the detected bioluminescence signal from the lung regions.

**Figure 5:**
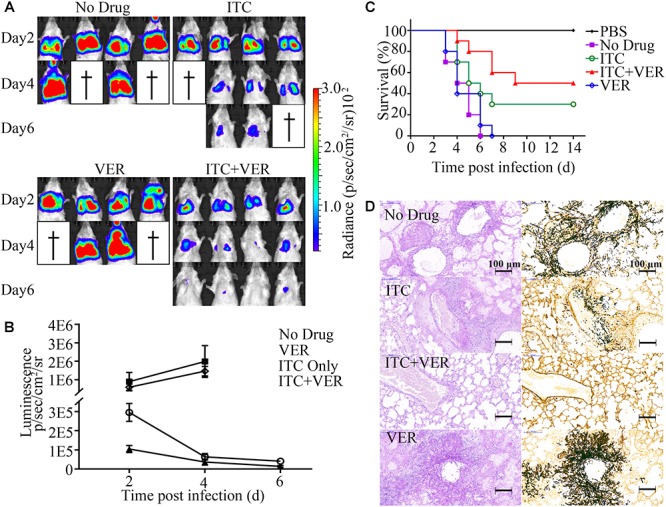
Coadministration of itraconazole and verapamil improves survival rate and reduces lung burden. **(A)** Luciferase signals from four infected mice each from the groups treated with ITC (20 mg/kg), VER (5 mg/kg) or ITC + VER were measured by live animal imaging. Luciferase activities at days 2, 4, and 6 post-infection are shown. **(B)** Luminescence acquired from the lung area of each mouse (in **A**) was analyzed with the software Living Image 4.2. Error bars indicate SD. **(C)** Ten infected BALB/c mice receiving ITC, VER or ITC + VER treatment were monitored for 14 days. A Kaplan–Meier survival plot is shown. **(D)** Lung tissues from mice of the No Drug, ITC, ITC + VER, and VER groups were isolated and stained with PAS and silver stain.

As shown in Figure 5A, after 2 days, luminescence signals were detected in the chest area in all mice but not in control mice (data not shown), indicating successful infection. Infected mice in the No Drug group showed an increase in bioluminescence signals accompanied by weight loss (see Supplementary Figure S3) and mouse death (Figure 5C) within 6 days. Approximately half of the mice succumbed to serious infection with high luminescence signals at day 4 post-infection. An almost identical result was observed in the VER monotherapy group compared to the No Drug group because no significant difference was found in luminescence signals, weight loss or mouse death.

In contrast, compared to the No Drug group, the ITC monotherapy group showed significantly decreased luminescence signals during the observation period, with improved survival (30% survival rate, relative to the No Drug group), indicating the effectiveness of the antifungal therapy. Notably, the luminescence signal indicating infection was further diminished under the combination therapy of ITC and VER compared to ITC monotherapy. The signal in the ITC plus VER group was reduced threefold compared to that of the ITC monotherapy group at day 2. Two animals recovered from the disease and showed almost no luminescence signals above background at day 6. With the decrease in signal, the weight gain started at day 6 and then increased and finally returned to prechallenge weight values. Moreover, combination therapy significantly reduced the mortality rates. Among a total of ten mice, 50% of mice receiving ITC plus VER treatment survived the infection until the experiment terminated, which altered the mouse survival under ITC monotherapy.

A corresponding decrease in mouse lung tissue damage was observed, as determined by PAS staining and silver staining of lung tissue sections (Figure 5D). Control mice in the No Drug group showed severe lesions in lung tissues characterized by multifocal inflammatory foci formed by neutrophils and macrophages containing *A. fumigatus* hyphae, which were centered on bronchioles and extended to alveoli. Pulmonary lesions in the VER groups were similar to the No Drug group. However, the overall hyphae and inflammatory foci were decreased under ITC monotherapy. Furthermore, the ITC plus VER group showed a dramatic decrease in pulmonary fungal burden compared to that under ITC monotherapy. Hyphae were barely observed in the ITC plus VER group, with almost normal lung tissue structure.

## Discussion

In this work, we present data demonstrating that: (1) the clinical commonly used antiarrhythmic drug VER works as a potentiator in azole susceptibility against *A. fumigatus in vitro* and *in vivo*. (2) VER blocks the calcium signaling pathway and decreases the transient calcium response induced by ITC in *A. fumigatus*. (3) VER may influence the drug pump activity in *A. fumigatus.* Moreover, we built a luciferase-probed murine pulmonary aspergillosis model to monitor the progress of infection and evaluate the drug efficacy of combination therapy.

### The Possible Mechanism of ITC-VER Synergism

In recent years, several investigators have tested the antifungal activity of VER against fungal isolates. VER was reported to impact the adhesion, hyphal development, gastrointestinal colonization and oxidative stress response of *C. albicans* (Yu et al., 2014a,b) and potentiate the antifungal effect of fluconazole or tunicamycin against *C. albicans* biofilm (Yu et al., 2013). However, in *A. fumigatus*, VER was reported to inhibit biofilm formation, but no detectable difference was found when treated with ITC (see Supplementary Figure S4) and antagonism with VRC (Nazik et al., 2017). Moreover, no antifungal activity of VER alone was observed in *A. fumigatus* (Afeltra et al., 2004; Homa et al., 2017), which was also observed in this study within the tested range (MIC > 9600 μg/ml). However, in the presence of VER, the MIC values of ITC decreased in most of tested isolates, which is comparable to previously reported observations (Homa et al., 2017).

Our results suggested that the specific function of VER as an ITC sensitizer may be explained by two hypotheses: (1) VER may block the calcium channel and then influence the calcium signal pathway, which is crucial in fungal adaption under a ITC environment. (2) VER may block the function of the drug efflux pump so that the intracellular effective ITC concentration is increased, resulting in an increase of ITC drug efficacy.

The putative Ca^2+^ channel system in *Aspergillus* species, namely, CchA and MidA, is similar in the topology to L-type voltage-gated calcium channel in higher eukaryotes (Wang et al., 2016). VER, a specific inhibitor of L-type voltage-gated calcium channel in eukaryotic cells that is used for treating clinical cardiac disease, is inferred to block the fungal calcium channel system as well. In this work, we provide direct evidence for the blockage efficacy of VER in fungal calcium signaling pathway. As shown in Figure 2, the maximum [Ca^2+^]_c_ amplitude with VER pretreatment in response to ITC stimulation decreased to 41% (Figure 2). The decrease [Ca^2+^]_c_ amplitude directly leads to the failure of calcium signal transmission, which is essential for fungal stress adaption and viability. Another interesting finding of the study was that: the resting level obviously increased following VER pretreatment, which was also observed in *Neurospora crassa* under VER or diltiazem treatment (Binder et al., 2010). In contrast, the resting level and amplitude of [Ca^2+^]_c_ under ITC stimulation with EGTA pretreatment was significantly decreased, as EGTA, a Ca^2+^ chelator, reduced the Ca^2+^ source from the origin. This increased resting level of [Ca^2+^]_c_ may be related to the continuous inhibitory effects of the influx and/or Ca^2+^ pumps or Ca^2+^ channel and/or the activity of antiporters located in the cell membrane or the membranes of the organelle for internal Ca^2+^ stores. However, further studies are warranted to clearly verify how VER influences Ca^2+^ entry in *A. fumigatus*.

Another possible explanation for the combination synergism is that VER influences the activation of the drug efflux pump. Efflux of the toxic drugs by membrane transporters is a predominant strategy for fungal cells to maximize their survival (El-Awady et al., 2016; Chowdhary et al., 2017). The ATP-binding cassette (ABC) transporters are membrane proteins that mediated efflux dominates (Ernst et al., 2005; El-Awady et al., 2016). In humans, VER is found to be a good inhibitor of both ABC transporters ABCB1 and ABCB4, and therefore increases intracellular drug concentrations (El-Awady et al., 2016). Recent studies have confirmed the potential value of VER in reversing the drug resistance of antitumor (McCarthy et al., 2014) or antituberculosis (Machado et al., 2016) drugs, for VER is a substrate of the mammalian or bacterial efflux pumps. In our study, VER decreased the efflux of the azole mimic drug Rh 6G in *A. fumigatus* cells (Figure 3). We suspect that VER may inhibit the transporters in drug efflux and thus increase intracellular concentrations of Rh 6G. Another possibility is that VER might affect mitochondrial respiratory function and produce mitochondrial dysfunction in the *A. fumigatus* strain, because Rh 6G is reported to reflect the mitochondrial function (Scaduto and Grotyohann, 1999). The mitochondrial dysfunction may affect the energy supply for the ATP-driven pump in drug efflux. Either way, we suggest that the observed synergism of combination maybe due to decreased ITC accumulation in combination result from the blockage of drug pump activity of fungal cells by VER.

The two hypotheses mentioned above also explain the results of the ergosterol test. As shown in Figure 4, further reduction of ergosterol was observed after VER addition compared to ITC alone, while VER alone displayed no effect on ergosterol. We suspect that the blockage of calcium signaling pathway by VER resulted in dysfunction of essential physiological activities including ergosterol synthesis under ITC environment. In addition, it is suggested that the inhibition of fungal drug efflux efficacy by VER resulted in an increase of intracellular ITC accumulation. The increased ITC concentration may bring further reduction of ergosterol biosynthesis. After all, ergosterol depletion in cell membranes and organelle membranes impacts membrane fluidity and enzyme function, which may alter cellular stress responses and threaten fungal survival (Alcazar-Fuoli and Mellado, 2012).

### *In vivo* Assay

The *in vivo* results in our studies reflected the *in vitro* data. Our findings suggest that ITC combined with VER showed an improved survival benefit in the invasive aspergillosis mouse model (Figure 5). The benefit, however, may not be achieved by increasing the dose of ITC alone. Previous studies have shown that the antifungal effect of ITC *in vivo* is not dose-dependent (Tansho et al., 2006). An increased dose of ITC does not always result in enhanced survival. Conversely, the use of long-term, high-dose azoles may lead to increased toxicity or side effects and the emergence of drug resistance. To some extent, co-administration may help reduce the needed dose of azoles, shorten the treatment period and reduce the occurrence of drug resistance as a result of the reduced use of azoles. However, the current study only suggests the potential value of ITC-VER combination. To evaluate the safety of the ITC-VER combination, further pharmacological experiments and cytotoxic tests are warranted.

Other reagents that affect microbial calcium ions have also been reported to impact azole sensitivity in models of *in vivo* candidiasis or aspergillosis. Gamarra et al. suggested that AMD, which affects calcium homeostasis and leads to an instant influx of calcium ions (Courchesne and Ozturk, 2003), decreased the virulence combined with fluconazole in a murine model of candidiasis. Liu et al. confirmed that EGTA displayed an adjunctive antifungal effect on ITC in a *Galleria mellonella* invasive aspergillosis model (Liu et al., 2016). Unlike these reagents, VER not only disturbs the calcium ion balance but also may block the drug pump function. Additionally, this is the first work showing the use of *in vivo* imaging technology to monitor the therapeutic effect of aspergillosis therapy combined with non-antifungal and antifungal drugs. Bioluminescence imaging of fungal infections is a noninvasive technique that allows real-time detection of infected lesions and fungal burden quantitation (Brock et al., 2008; Avci et al., 2018). Conventional mouse models for aspergillosis are sacrificed at several time points to confirm the progression of infection by histopathology or CFU, which consumes more animals and more time than *in vivo* imaging (Savers et al., 2016). The value of bioluminescence imaging in monitoring and comparing the efficacy of antifungals against *Aspergillus* strains was evaluated in previous work (Galiger et al., 2013). In our study, an evident increase in the light emission correlated with animal death and pathological damage, and a decrease in the light emission correlated with better outcomes of infected mice. Therefore, our study suggests that bioluminescence imaging may provide valuable information to evaluate the fungal virulence or the efficacy of drug combinations in mice.

## Data Availability

The raw data supporting the conclusions of this manuscript will be made available by the authors, without undue reservation, to any qualified researcher.

## Ethics Statement

This study was carried out in accordance with the recommendations of the Guide for the Care and Use of Laboratory Animals of the National Institutes of Health, United States. The protocol was approved by the Animal Care and Use Program at Nanjing Normal University, China (protocol number 2090658).

## Author Contributions

QZ carried out the experiments, analyzed the data, and wrote the manuscript. ZZ and NL assisted in the animal assays. PC assisted in the *in vitro* assays. LL and HS contributed to the manuscript revision and overall support.

## Conflict of Interest Statement

The authors declare that the research was conducted in the absence of any commercial or financial relationships that could be construed as a potential conflict of interest.
