# Supplementary material for: In vitro and in vivo Efficacy of a Synergistic Combination of Itraconazole and Verapamil Against Aspergillus fumigatus
Source: Front Microbiol. 2019 Jun 7;10:1266. doi: 10.3389/fmicb.2019.01266 (PMC6567931; doi:10.3389/fmicb.2019.01266)
Supplement: Supplementary file 1 [file Data_Sheet_1.pdf]

Supplement data

Supplementary Fig S1 VER improves the growth inhibition of ITC on agar plates against *A. fumigatus.*

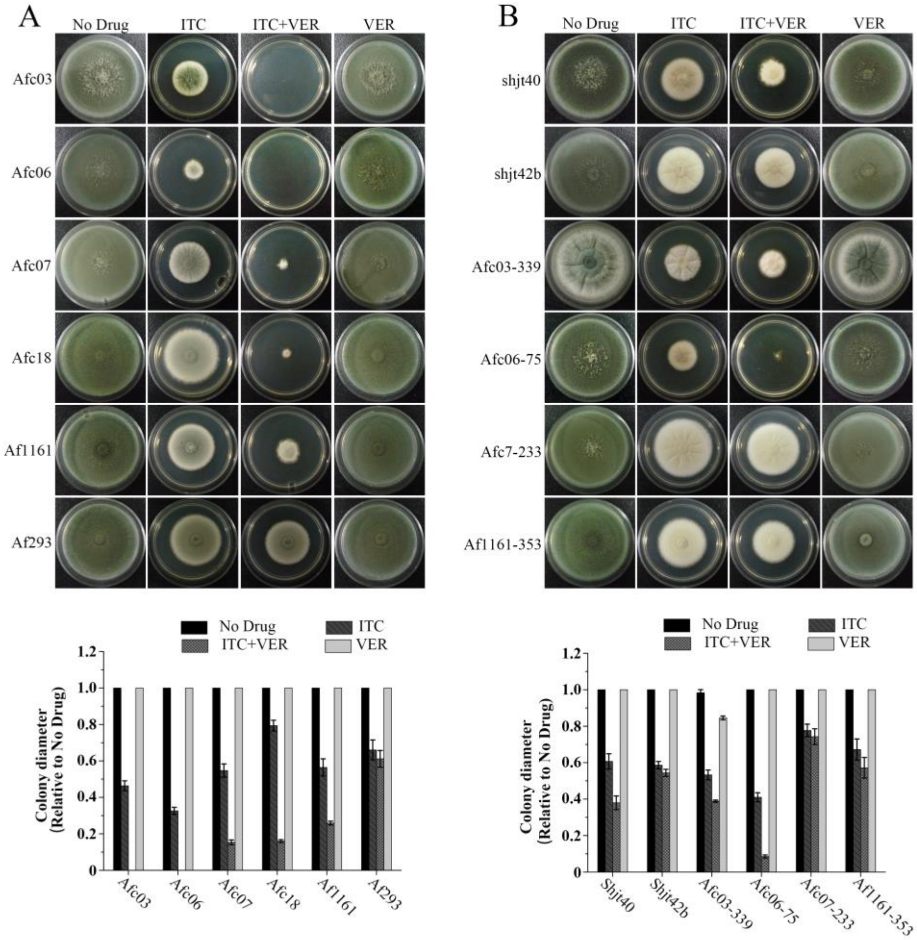

Testing of growth inhibition on agar plates by itraconazole (ITC) (0.1 μg/ml) with verapamil (VER) (200 μg/ml for A and 600 μg/ml for B) against ITC-susceptible (A) and ITC-resistant (B) *A. fumigatus* isolates.

Supplementary Table S1 Drug interaction evaluation of *A. fumigatus* strains.

Strains	MIC (µg/ml)				ITC-VER Interaction	
	Single		Combined			
	ITC	VER	ITC	VER	FICI	Interpretation
Af1161	0.25	>9600	0.0625	40	0.254	SYN
Af1161-AEQ	0.25	>9600	0.0625	40	0.254	SYN
Af1161-Luc2	0.25	>9600	0.0625	40	0.254	SYN

Supplementary Fig S2 Bioluminescence of the luciferase reporter strains.

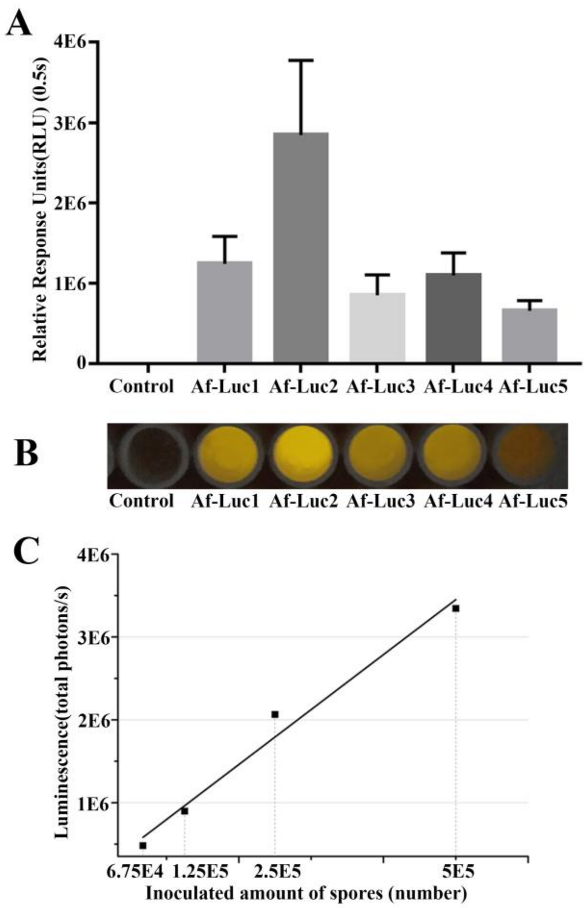

A and B. Comparison of light emission (A) and visual observation (B) of Af1161 (as control) and five reporter *A. fumigatus* strains carrying the codon-optimized luciferase gene. The respective strain was inoculated in a white 96-well plate with 2.5×10^5 conidia for 12 h at 37°C. Luminescence was assayed using a microplate reader after adding D-luciferin. Average values of three independent replicates are shown with SD error bars.

C. Correlation of bioluminescence and inoculated amount of Af-Luc2 spores using the IVIS Lumina XR system. Average values of three independent replicates are shown with SD error bars.

Supplementary Fig S3 Body weight monitoring of therapy efficacy.

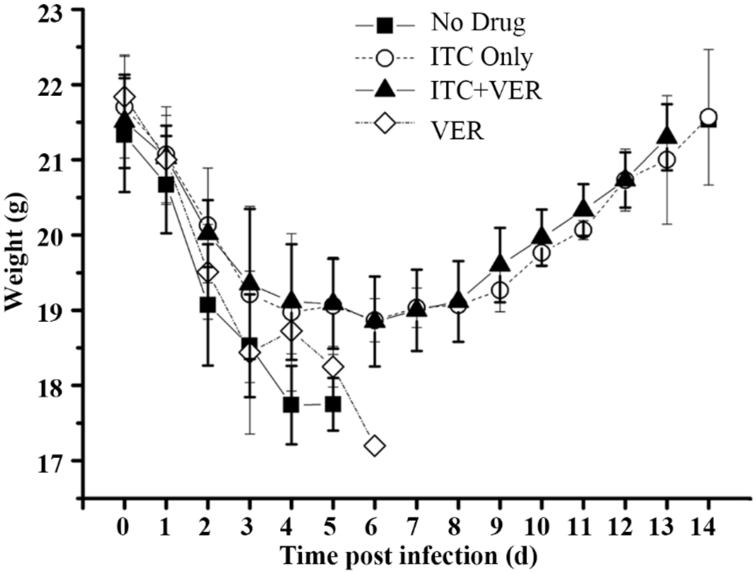

Average values (N=10) are shown with SD error bars.

Supplementary Fig S4 Effect of ITC and VER (μg/ml) on inhibition of biofilm formation of Afc03 strain.

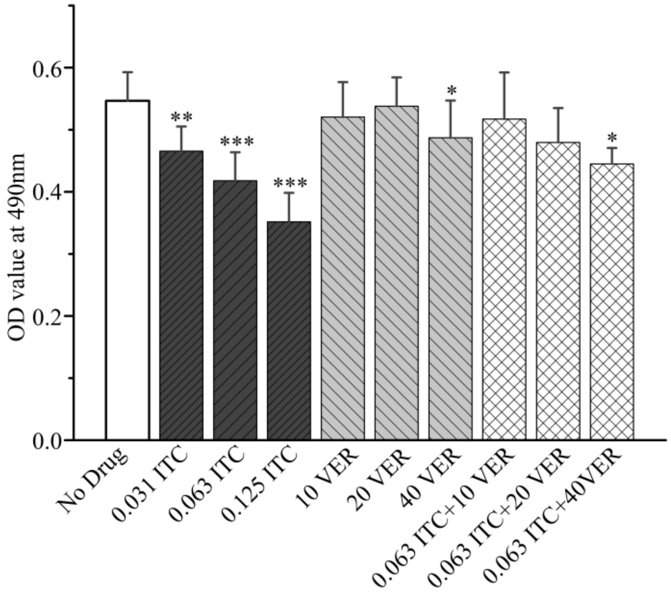

ITC alone was inhibitory at 0.031-0.125 μg/ml compared to the No Drug control (left bar).(**, $p< 0.01$, ***, $p< 0.001$). VER(40 μg/ml) alone inhibited *Aspergillus* biofilm formation (seventh bar from left) (*, $p< 0.05$). ITC (0.063 μg/ml)+VER (40 μg/ml) was also inhibitory, as shown in tenth bar from left (*, $p< 0.05$). However, no detectable difference was found in VER (40 μg/ml) alone and ITC (0.063 μg/ml)+VER (40 μg/ml).
